# Indwelling pleural catheter tract metastasis from renal cell carcinoma

**DOI:** 10.1002/rcr2.527

**Published:** 2020-02-03

**Authors:** See‐Wei Low, Kenneth K. Sakata

**Affiliations:** ^1^ Pulmonary and Critical Care Mayo Clinic Arizona Phoenix AZ USA

**Keywords:** Catheter tract metastasis, indwelling pleural catheter, renal cell carcinoma

## Abstract

This case highlights the importance of recognizing any new soft tissue abnormalities in cancer patients with an indwelling pleural catheter (IPC) or who has had an IPC. This report also describes the first case of catheter tract metastasis (CTM) due to renal cell carcinoma (RCC) and the second case of CTM post‐IPC removal.

## Graphical abstract

The importance of recognizing new nodules or subcutaneous mass in patients who has had indwelling pleural catheter (IPC) or overlying the IPC site is highlighted. This report describes the first case of catheter tract metastasis (CTM) due to renal cell carcinoma (RCC) and the second case of CTM post‐IPC removal.

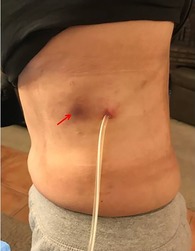

## Clinical Image

**Figure 1:**
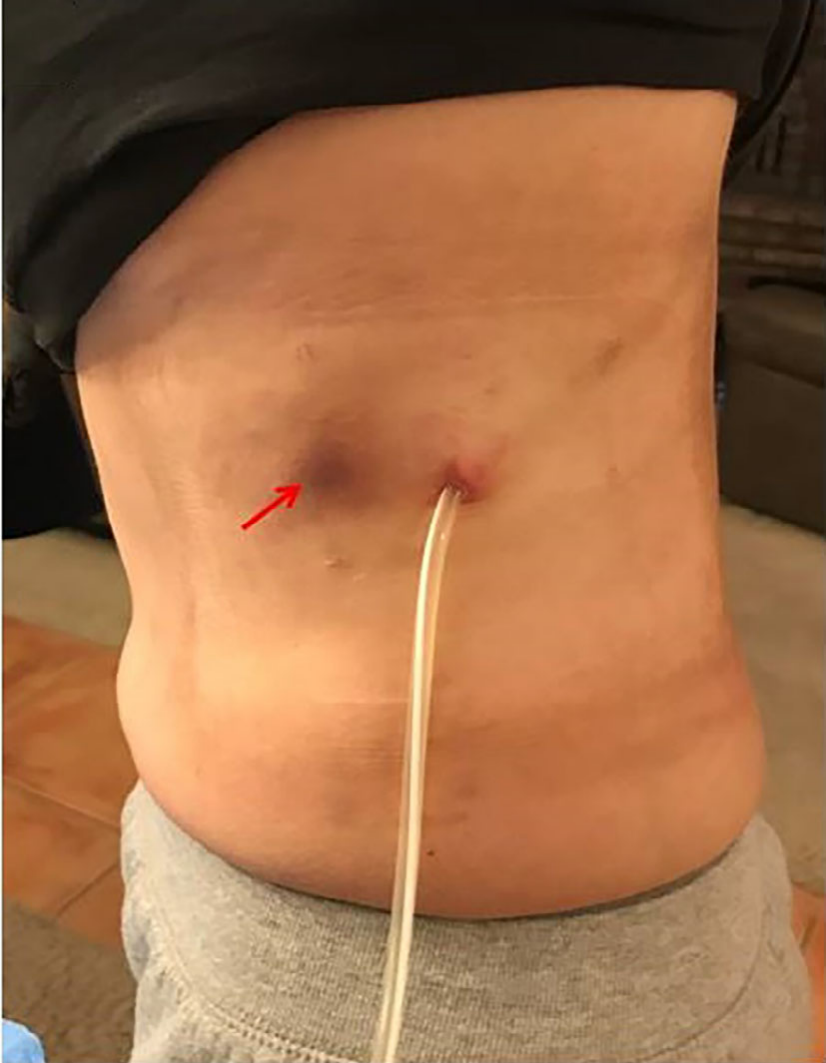
Skin discolouration along her previous tunneled tract 10 months after her first IPC placement (highlighted with red arrow).

**Figure 2:**
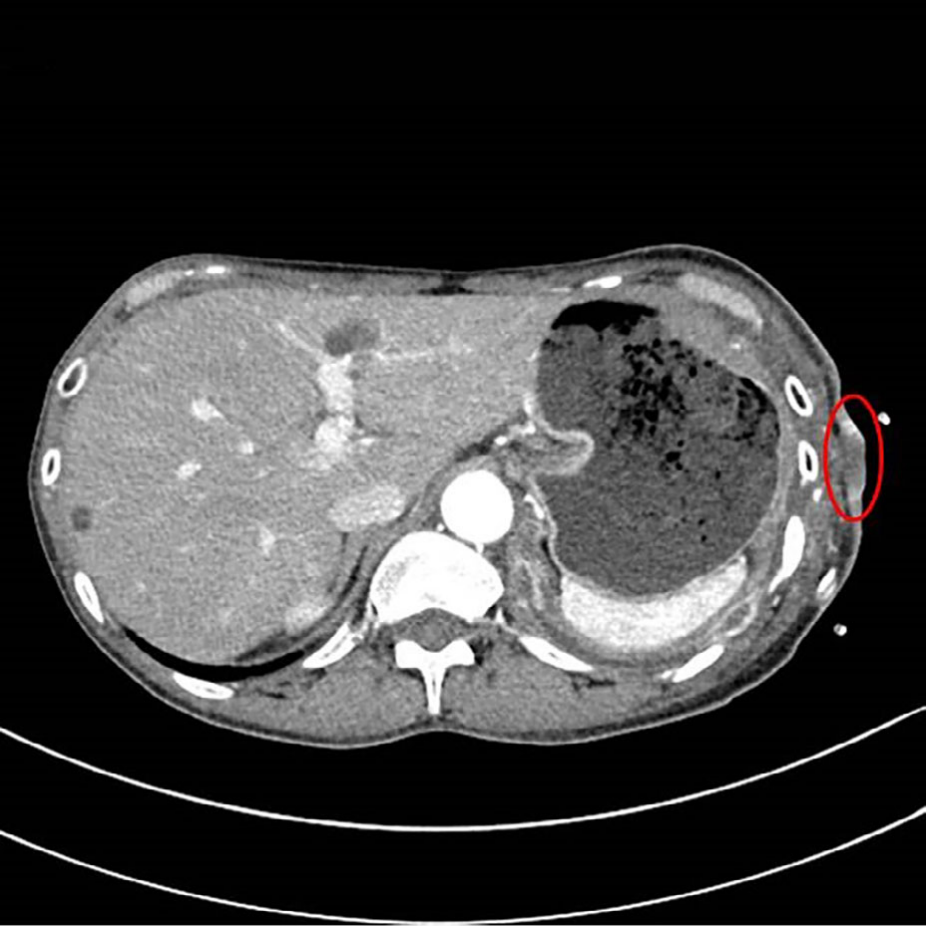
Computed tomography of the chest showing the presence of a soft tissue mass protruding out of the subcutaneous tissue through the skin on the left (highlighted with red oval).

**Figure 3:**
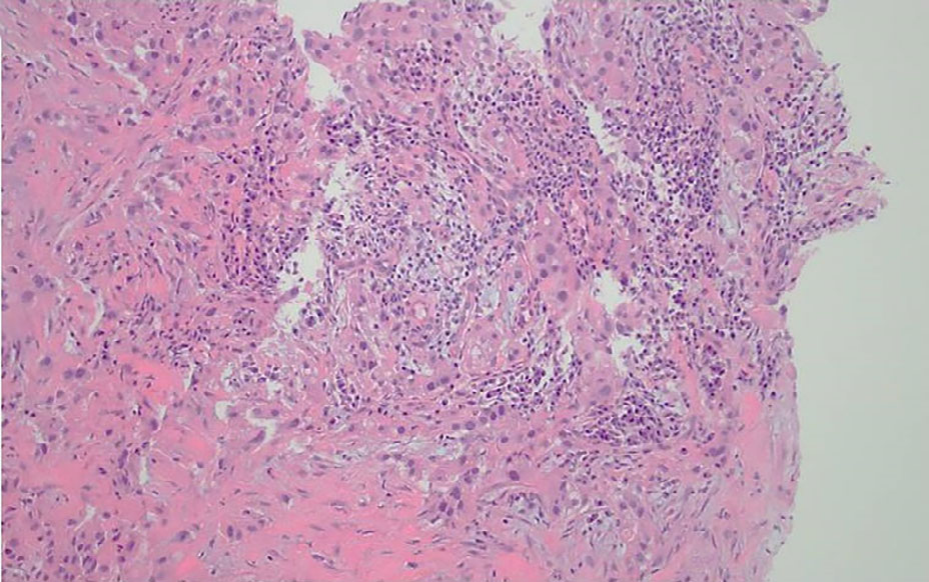
Histological confirmation of metastatic renal cell carcinoma from the biopsy of the left subcutaneous chest wall mass.

A 54‐year‐old female had a left indwelling pleural catheter (IPC) placed in March 2017 for a recurrent pleural effusion from renal cell carcinoma (RCC). She achieved spontaneous pleurodesis within two months and her IPC was removed. Her pleural effusion recurred four months later and a second IPC was placed at a separate site, posterior to her initial IPC placement. In January 2018, 10 months after her first IPC placement (eight months after her first IPC was removed), despite targeted treatment with lenvatinib and everolimus, she noticed an area of skin discolouration associated with pain along the previously tunneled tract of her first IPC (Fig. 1). A computed tomography (CT) of the chest showed presence of a left lateral chest wall mass (Fig. 2). An ultrasound‐guided biopsy of the chest wall mass revealed metastatic RCC (Fig. 3), confirming catheter tract metastasis (CTM). The patient received a total of 30 Gy in 15 fractions of external beam radiation to her left chest wall and back. Upon completion of therapy, repeat CT chest showed improvement of the left lateral chest wall mass and pleural metastases. CTM is a rare complication of IPCs. It often presents with a new and painful subcutaneous nodule/mass overlying the IPC insertion site or its tunneled subcutaneous tract. The diagnosis of CTM is made on clinical and radiological grounds with use of histological confirmation if needed. CTM often occurs late, at a median of 280 days post‐IPC placement.

### Disclosure Statement

Appropriate written informed consent was obtained for publication of this case report and accompanying images.

